# Long-Acting Injectable Antipsychotics: A Systematic Review of Their Non-Systemic Adverse Effect Profile

**DOI:** 10.2147/NDT.S309768

**Published:** 2021-06-14

**Authors:** Monica Zolezzi, Rawan Abouelhassan, Yassin Eltorki, Peter M Haddad, Mahtab Noorizadeh

**Affiliations:** 1 College of Pharmacy, QU Health, Qatar University, Doha, Qatar; 2 Mental Health Hospital, Hamad Medical Corporation, Doha, Qatar

**Keywords:** intramuscular preparations, depot antipsychotics, injection site adverse effects

## Abstract

**Introduction:**

Long acting injectable (LAI) antipsychotics are commonly used in the treatment of schizophrenia to improve adherence and clinical outcomes. Concerns have been reported in relation to their non-systemic or injection site adverse effect profile. As such, this study aims to review and evaluate all evidence reporting injection site adverse effects with LAI antipsychotics.

**Methods:**

An electronic search was systematically conducted through four databases (PubMed, Embase, SCOPUS, Cochrane) in order to identify studies investigating injection-site reactions associated with LAI antipsychotics. Unpublished studies such as conference proceedings and clinical trial registries were also searched. The search was limited to literature published in English without year limits.

**Results:**

Of a total of 189 citations that were identified from the electronic database search, 12 were selected for inclusion in this review. Various injection site reactions were reported in these studies, including pain, bleeding, and swelling. Overall, the studies reported a low incidence of these injection site reactions. Only a minority of the included articles compared injection site reactions between different LAI antipsychotics.

**Conclusion:**

Injection site pain was the most commonly reported injection site adverse effect across all articles reviewed. The low incidence of injection site adverse effects associated with LAI antipsychotics indicates that these formulations appear to be well tolerated by patients. More head-to-head trials comparing second generation LAI antipsychotics are needed.

## Introduction

Long acting injectable (LAI) antipsychotics (also referred to as depot antipsychotics) are concentrated formulations which, following intramuscular injection, release the antipsychotic drug slowly over time. This allows an effective maintenance dose of the antipsychotic to be delivered with injections at intervals that range from 2 weeks to up to several months.^1^ Although uncertainty exists regarding the overall benefits of LAI antipsychotics over oral administration, there is growing evidence of their effectiveness in preventing relapse and rehospitalization, and in decreasing the negative consequences of poor adherence during the early phases of schizophrenia.^2–6^ The slow release of the antipsychotic from the injection site, and long half-life, reduces the risk of an abrupt loss of efficacy if a dose is missed.^7^ Although non-compliance with antipsychotic medication has many underlying causes (eg, intolerability with side effects, forgetfulness, and stigma associated with taking oral medications), it is the most common reason to start LAI antipsychotics.^8–10^ Other reasons include patient convenience and adequate alternative for those who have difficulty ingesting or absorbing oral formulations. A large systematic review and meta-analysis revealed that antipsychotics when administered in a LAI formulation did not differ on all serious systemic adverse events than when administered orally.^11^ However, a range of potential non-systemic or injection site reactions may also occur with LAI antipsychotics, such as injection site pain, skin thickening, infection, erythema, nodules, lumps, bleeding, and tenderness.^10^

First-generation LAI antipsychotics consist of the antipsychotic esterified to a decanoate, which is dissolved in an oily vehicle.^12^ A variety of delivery systems have been employed for the second-generation antipsychotics (SGAs) LAIs.^1^ Risperidone, for example, is encased in degradable polymer microspheres. The other SGA LAIs (olanzapine pamoate, paliperidone palmitate, and aripiprazole) consist of microcrystalline salts of the antipsychotic in an aqueous suspension. These different delivery systems may have a role on the prevalence and the severity of injection site adverse effects. It has been reported that first-generation LAI antipsychotics may be associated with a higher rate of pain and injection site reactions due to the oily vehicle they are administered in.^12–15^ Frequent large volume administrations of these oil-based injections have also been reported to be associated with the development of muscle fibrosis and granuloma.^15^

Considering the relatively limited attention that has been paid to non-systemic side effects associated with LAI antipsychotics, and which are rarely reported in clinical trials, this study aims to systematically review all the available evidence reporting injection-site adverse effects associated with the administration of LAI antipsychotics. This review of local adverse events of single LAI antipsychotics will provide evidence-based recommendations for their safe use in practice and may help clinicians in tailoring their choice of treatment.

## Method

Based on a pre-determined protocol (Appendix 1), which outlined the different steps of this systematic review of the literature, an electronic database search was conducted from database inception using PubMed, SCOPUS, Embase and Cochrane databases in order to identify studies investigating injection-site reactions associated with LAI antipsychotics. Search terms included “LAIAPs”, “long acting depot”, “antipsychotics”, “neuroleptics”, “local reactions” and “injection site adverse effects”. To avoid publication bias, unpublished studies such as conference proceedings and clinical trial registries were also searched. In addition, a quick update of the literature was undertaken to identify articles published between the time of the initial search and the time of publication.

**Figure 1:**
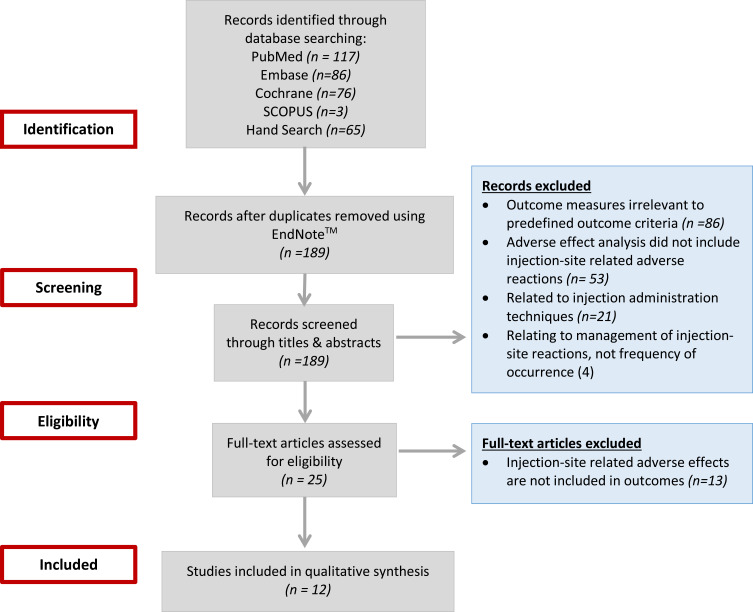
Progress flow chart of literature search and selection of articles for review.

The initial screening of article titles and abstracts was conducted by one reviewer (R.A.). Studies were selected for full review based on the following inclusion criteria: articles in English that investigated injection-site reactions associated with LAI antipsychotics or studies reporting any form of adverse effects relating to injection-site administration of antipsychotics. By injection site adverse effects, only local reactions of the skin and underlying tissue were considered. As such, non-localized adverse effects resulting from the intramuscular administration of the antipsychotic (such olanzapine-induced post-injection delirium/sedation syndrome) were excluded. Articles were also excluded if injection-site related adverse effects were not included in the study outcomes. The full text of all potentially relevant articles were retrieved and distributed among three reviewers (R.A., M.Z., Y.E.) to confirm eligibility. Figure 1 illustrates the progress flow chart of the literature search and selection of articles for synthesis. Key data including injection site adverse effects reported with different doses or dose-intervals of the same LAI antipsychotic and a comparison of adverse effects between different LAI antipsychotics was extracted from the articles and summarized on a data collection sheet using Excel.

The quality of the articles was assessed using the Critical Appraisal Skills Programme (CASP) Systematic Review checklist.^16^ At least two reviewers independently evaluated the articles for risk of bias. As recommended by the CASP appraisal tool, a scoring system was not used, and an overall assessment of bias was made. Depending on the study type, the corresponding CASP tool designed for each specific study type was utilized for the analysis, ie, randomized controlled trials (RCTs), cross-sectional studies and systematic reviews (SRs) and meta-analyses. Studies at high or unclear risk of bias may have overestimated or underestimated the results. We have included the number of “yes” criterion as a general gauge from each CASP tool for each respective study type ranging from 0 to 10, 0 to 12 and 0 to 11 for SRs, cross-sectional studies and RCTs, respectively. Disagreement was adjudicated by consensus.

## Results

Of the 189 citations identified in the initial electronic database search, 25 full-text articles were selected to undergo a more comprehensive review based on the inclusion/exclusion criteria. However, following the full-text review, 13 articles were excluded because these articles did not provide information on injection-site adverse effects. Therefore, a total of 12 articles were selected for full review and data extraction using a standardized data collection tool.^11^,^17–27^ Table 1 provides general information about these studies, their design and the outcomes that were reported. Table 2 lists the different LAI antipsychotics investigated in the 12 articles included in this review, and provides a summary of the associated injection-site reactions that were reported.^11^,^17–27^ Various injection site reactions for different LAI antipsychotics were reported in these studies, including pain, bleeding, and swelling. The most common injection site reaction was pain which was mainly reported in the studies by Hay,^17^ Jones et al,^18^ Atkins et al,^23^ and Kern Sliwa et al^26^ (Table 2). The majority of the studies presented comparative data among different LAI antipsychotics. The study by Kern Sliwa et al

**Table 1:** Details of Included Studies

Study #	Authors (Year)	Study Design	Sample Size (N)	LAI Antipsychotics Investigated	Outcomes Measured	Quality Score*
1	Hay (1995)^17^	Cross-sectional	224	FGAPs including: Haloperidol Zuclopenthixol Fluphenazine Flupenthixol	Incidence of injection site reactions using VAS Pain Bleeding Hematoma Leakage Indurations Transient nodules	8
2	Jones et al (1998)^18^	Cross-sectional	318	Haloperidol Flupenthixol	Prevalence of Injection site reactions Skin thickening Infection/erythema Nodules/lumps Bleeding Pain Tenderness Patient self-assessed reactions using a four-point likert scale 0 (none) to 4 (severe)	10
3	Lindenmayer et al (2005)^19^	Analysis report of 2 RCTs	1164	Risperidone 25mg, 50mg or 75mg q2w	Injection site reactions Pain Redness Swelling Induration VAS for patient pain assessment Investigators assessed injection site reactions by observation mainly assessing redness, swelling and induration	9
4	Nasrallah et al (2010)^20^	RCT	514	Paliperidone palmitate 25, 50 or 100mg q1M	Incidence of injection site reactions using Investigator and patient-assessed VAS Pain Redness Induration Swelling	11
5	Quiroz et al (2011)^21^	RCT	223	Risperidone 37.5mg or 50mg q2w	Incidence of injection site reactions Pain Redness Swelling Tenderness Induration Patient-rated VAS Investigators assessed reactions 30 minutes before risperidone LAI and 2 hours post-injection using a rating scale of 0–3 (0=absent, 1=mild, 2=moderate, and 3=severe)	10
6	Kane et al (2012)^22^	RCT	403	Aripiprazole q1M	Incidence of injection site reactions using VAS Pain Swelling Redness Indurations	11
7	Atkins et al (2014)^23^	Pooled analysis of 7 clinical trials	2399	Olanzapine 45–405 mg injection at 2-, 3- or 4-week intervals	Incidence of patient-assessed injection site reactions using VAS: Pain, reaction, mass, induration, nodule, irritation, abscess, hemorrhage, warmth, erythema, discoloration, extravasation, edema, paresthesia, rash	10
8	Kisely et al (2015)^24^	SR and MA	3994	Fluphenazine q2w or q1M Paliperidone q1M Risperidone q2w Olanzapine q2w Haloperidol q1M	Incidence of injection site pain using VAS in most of the studies included in the review Method of injection site evaluation was not disclosed for all studies	10
9	Chen et al (2016)^25^	Cross-sectional	434	FGAPs Risperidone	Incidence of injection site pain using patient-rated VAS	12
10	Misawa et al (2016)^11^	SR and MA	4902	Paliperidone Zuclopenthixol Risperidone Olanzapine Fluphenazine	Incidence of any local reaction at injection site (no further details)	10
11	Kern Sliwa et al (2018)^26^	RCT	1429	Paliperidone q1M vs q3M	Incidence of injection site reactions: Pain Indurations Erythema Swelling Injection site assessed within 30 minutes after each administration Visual analog scale (VAS) used for patient self-assessment Investigators assessed induration, redness, and swelling on a 4-point scale (0=absent; 1= mild; 2=moderate; 3=severe)	11
12	Ting et al (2019)^27^	SR and MA	4482	Olanzapine q2w or q1M Paliperidone q1M or q3M Fluphenazine Aripiprazole q1M Risperidone q2w	Incidence of injection site pain using VAS in most of the studies included in the review Method of injection site evaluation was not disclosed for all studies	10

**Note:** *As per the CASP tool.^16^

**Abbreviations:** RCT, randomized controlled trial; VAS, visual analog scale; q1M, once-monthly; q3M, every 3-months; q2w, every 2 weeks; FGAP, first generation antipsychotics; SR & MA, systematic review & meta-analysis.

**Table 2:** Key Findings Reported on the Selected Studies

Study #	Authors (Year)	LAI Antipsychotics Investigated	Key Findings
1	Hay (1995)^17^	FGAPs including: Haloperidol Zuclopenthixol Fluphenazine Flupenthixol	19.3% of patients experienced local reactions. 84 acute problems were reported: 31 of unusual pain, 21 of bleeding or hematoma, 19 of leakage of the drug from the injection site, 11 of acute inflammatory indurations, 2 of transient nodule formation.
2	Jones et al (1998)^18^	Haloperidol Flupenthixol	Clinically significant depot site reactions were pain (8.2%), bleeding (6.9%), nodules (4.4%) and skin thickening (3.5%). Absence of clinically significant erythema. Haloperidol was associated with the highest proportion of site reactions; not significantly different from other LAI antipsychotic preparations.
3	Lindenmayer et al (2005)^19^	Risperidone 25mg, 50mg or 75mg q2w	Mean VAS scores at the first and final injection showed no significant difference between different doses of risperidone. Ratings indicated high patient tolerance throughout the trial (baseline=7.3; endpoint=7.7; p<0.0001 versus baseline).
4	Nasrallah et al (2010)^20^	Paliperidone palmitate 25, 50 or 100mg q1M	Injection site pain was reported by 14% for patients (12% mild; 2% moderate to severe). Redness, induration, or swelling were infrequent, generally mild, and decreased over time.
5	Quiroz et al (2011)^21^	Risperidone 37.5mg or 50mg q2w	7 of the 51 patients who received at least two deltoid injections discontinued the treatment. None of the discontinuations were due to injection-site related reasons.
6	Kane et al (2012)^22^	Aripiprazole q1M	Mean intensity of injection site pain was minimal; reductions of VAS scores were seen between the first and last injection administered (6.1) and (4.9), respectively. Redness and swelling was absent in 73.8–95.0% of patients. Injection site induration significantly reported more frequently than placebo (1.9%).
7	Atkins et al (2014)^23^	Olanzapine 45–405 mg injection at 2-, 3- or 4-week intervals	Pain was the most commonly reported, occurring in 2.9% of patients All other types of reactions occurred in <1% of patients Rates of discontinuation were low
8	Kisely et al (2015)^24^	Fluphenazine q2w or q1M Paliperidone q1M Risperidone q2w Olanzapine q2w Haloperidol q1M	Q2w injections were significantly less likely to lead to pain (RR=0.16, 95% CI=0.07–0.38; 2 studies n= 1667). Risperidone q2w was less likely to lead to site pain than q1M paliperidone (not statistically significant)
9	Chen et al (2016)^25^	FGAPs Risperidone	FGAPs showed lower injection pain severity compared to patients receiving risperidone LAI (VAS ratings), P<0.05
10	Misawa et al (2016)^11^	Paliperidone Zuclopenthixol Risperidone Olanzapine Fluphenazine	The prevalence of injection site reactions was not significantly different between the various antipsychotic formulations investigated
11	Kern Sliwa et al (2018)^26^	Paliperidone q1M vs q3M	Spontaneously-reported injection site pain for PP3M vs PP1M (3% vs 2%). Investigator-rated injection site reactions for PP3M vs PP1M: Induration (12% vs 10%), redness (10% vs 9%), swelling (7% vs 9%).
12	Ting et al (2019)^27^	Olanzapine q2w or q1M Paliperidone q1M or q3M Fluphenazine Aripiprazole q1M Risperidone q2w	Q2w injections were less likely to cause injection site pain than q1M injections (finding from sensitivity analysis).

**Abbreviations:** q1M, once-monthly; q3M, every 3-months; q2w, every 2 weeks; FGAPs, first generation antipsychotics.

(2018)^26^ compared the occurrence of injection site reactions with paliperidone once-monthly (PP1M) and every 3 months (PP3M). Injection site reactions and pain were infrequent, low and mild, and were similar between the PP1M and the PP3M, regardless of the dose and of the volumen or location of the injection. The systematic reviews and meta-analyses conducted by Kisely et al^24^ and Ting et al^27^ comparing several types of LAI antipsychotics, reported that 2 weekly administration was less likely to lead to injection site pain than 4 weekly administration. The studies by Hay^17^ and Jones et al^18^ both reported clinically significant reactions such as pain, bleeding, nodules, thickening, hematoma and indurations primarily associated with the administration of haloperidol decanoate, although these were not significantly different from those reported with other depot antipsychotics. Patient satisfaction and subjective well-being were investigated in the studies by Lindenmayer et al^19^ and Chen et al,^25^ where ratings indicated significantly high patient satisfaction with LAI administration (p<0.05). Overall, the reported incidence of these reactions was relatively low or mild in the included studies.

The quality assessment scores reported in Table 1 indicate that the majority of the studies met all the required criteria provided by the quality assessment tool. The studies by Quiroz et al^21^ and Chen et al^25^ had lower scores due to the absence of accurate measurement of exposure and outcome (Chen et al^25^ study) and due to the open-label nature of the Quiroz et al^21^ study. The study by Jones et al^18^ did not meet most of the CASP criteria for not taking into consideration confounding factors throughout the analysis. Similarly, the score of the study by Hay^17^ was not high because confounding factors were not taken into consideration during the analysis, and also because several details in the methods were not disclosed, such as statistical analysis and whether patients were monitored adequately in terms of specific follow-up measurements required.

## Discussion

In this study, a systematic review was conducted on all the available evidence reporting injection-site adverse effects associated with the administration of LAI antipsychotics aiming to provide evidence-based recommendations for their safe use in practice. The most commonly reported injection site reaction across the 12 articles included in this study was pain at the injection site. In an indirect comparative study of injection-site pain associated with first-generation LAI antipsychotics versus paliperidone palmitate (PP), researchers suggested that PP may be associated with lower mean pain severity than first-generation products.^26^ Lindenmayer and colleagues also found LAI risperidone less painful than pain associated with first-generation antipsychotics.^19^ Authors attributed this finding to the aqueous based formulations of the second generation antipsychotics. The lack of head-to-head comparisons of injection-site pain among the second generation LAI warrants further investigation. Other reasons for injection pain have been attributed to utilization of an inappropriate length of needle, inadequate skills, and inappropriate administration techniques which may cause the medication to enter the subcutaneous tissues instead of the targeted intramuscular site, resulting in persisted medication release for a longer duration of time.^28^ This, in turn, causes irritation, inflammation, and pain.^10^ When administering first generation LAI antipsychotics, it has long been recommended to use the Z-track technique, as it prevents leakage from injection sites and reduce the incidence of injection site adverse effects.^6^ However, in a study by Lin et al, researchers compared pain associated with first-generation LAI antipsychotics using three different intramuscular techniques (air-bubble, z-track, and a combination of both), and found no difference in the pain level between the three types of injection methods.^29^ There was no detailed description of the injection administration techniques used throughout the studies included in this review, therefore it is difficult to judge if any particular administration technique was more favorable in regards to pain or other injection site reactions. Literature supports rotating injection sites, avoiding excessive injection volume, and increasing injection intervals to minimize overall injection site side effects and improve patient acceptability of LAI antipsychotics.^15^ The specific muscle in which the LAI antipsychotic is administered may also influence injection site pain as well as patient acceptability. It is now recommended that LAI antipsychotics are administered into the ventrogluteal muscle (side of hip), as the traditional dorsogluteal site carries additional risks due to the proximity of the sciatic nerve.^30^ The deltoid muscle, which is used for administering the LAI PP and risperidone, have the advantage of being the most accessible for administration and is often preferred by patients as it is viewed as less intrusive than the gluteal injection site.^13^ The study by Hay^17^ concluded that the effects of repeated injections of high doses over many years, along with the irritant properties of the drug, contributed to the development of site reactions. The study by Ting et al^27^ suggested that the incidence of injection site pain may decrease as the number of injections received increases, possibly because it has been observed that patients become desensitized to the injections when used over a long period of time.^31^ In the study by Jones et al^18^ a clinically significant increase of site reactions was reported with higher concentrations of LAI antipsychotics among patients who had been receiving more frequent injections, and a significantly higher volume of depot administered in the previous 12 months. However, when adjusting for the volume of depot administered, the relationship between the severity of the reactions and the formulation concentration was no longer significantly different. This reveals that such injection site reactions may be prevented and reduced by perhaps increasing the interval between injections and using low volume (of more concentrated) preparations.

Most studies investigated in the current systematic review were of relatively high quality (Table 1). All the studies included used a patient (self-assessment) tool to report the outcomes related to LAI antipsychotics administration. The utilization of a self-assessed method to report injection site adverse effects is considered relevant and appropriate in relation to the study purpose since pain for example, is a subjective sensation that can only be reported by the patients themselves. Other adverse effects that can be assessed visually, such as rashes, bleeding or indurations, were reported by the investigators in all studies included in this review. In addition, the article by Kern Sliwa et al^26^ ensured that those patients included did not receive long-term administration of LAI antipsychotics within at least 4 weeks of enrollment while the remainder of the studies did not determine a specific duration of previous depot antipsychotics administration.

In conclusion, the majority of the articles discussed in this review reported that patients tolerate LAI antipsychotics formulations relatively well. The most commonly reported injection site reaction across all the studies was pain at the injection site, especially with oil-based formulations of LAI antipsychotics. Several strategies have been recommended to minimize injection site pain, such as selecting appropriate injection administration techniques, using suitable needle specifications, increasing the interval between injections and administering low volume of highly concentrated formulations of LAI antipsychotics. Noticeably, such strategies should be further investigated in order to assess their influence on improving injection-site reactions. Similarly, the selection of the muscle site for injection administration is an important consideration that is worth exploring further. Although the gluteal region is the preferred site for administering depot antipsychotics, the deltoid area is less intrusive for patients; hence, this may influence patient acceptability and compliance.^21^,^32–34^

## Study Limitations

Although this review generated important findings, some limitations in the literature review process need to be highlighted. First, the article by Kern Sliwa et al^26^ ensured that patients included did not receive LAI antipsychotics within at least 4 weeks of enrollment while the remainder of the studies did not clarify duration of any prior LAI antipsychotic administration. This is a limitation since the occurrence of injection site reactions may be influenced by the prolonged duration of LAI antipsychotic administration prior to the respective study. Second, the studies thus far available indicate that pain is less severe with second-generation LAI antipsychotics. However, there are very few head-to-head trials of different LAI antipsychotics. Thus, more head-to-head high-quality trials are required to confirm these injection site reactions with the different formulations used in second-generation LAI antipsychotics. Third, as there may have been relevant studies that did not produce positive results, publication bias is a possibility.
